# Long-term Reprogramming and Altered Ontogeny of Classical Monocytes Mediates Enhanced Lung Injury in Sepsis Survivor Mice

**DOI:** 10.1101/2025.05.16.654442

**Published:** 2025-05-21

**Authors:** Scott J. Denstaedt, Breanna McBean, Alan P Boyle, Brett C. Arenberg, Matthias Mack, Bethany B. Moore, Michael W. Newstead, Benjamin H. Singer, Jennifer Cano, Hallie C. Prescott, Helen S. Goodridge, Rachel L. Zemans

**Affiliations:** 1 Division of Pulmonary and Critical Care Medicine, Department of Internal Medicine, University of Michigan. Ann Arbor, Michigan, USA.; 2 Human Genetics, University of Michigan, Ann Arbor, Michigan, USA.; 3 Computational Medicine and Bioinformatics, University of Michigan, Ann Arbor, Michigan, USA.; 4 Department of Nephrology, University Hospital Regensburg, Regensburg, Germany; 5 Department of Microbiology and Immunology, University of Michigan, Ann Arbor, Michigan, USA.; 6 VA Center for Clinical Management Research, Ann Arbor, MI, USA; 7 Board of Governors Regenerative Medicine Institute and Department of Biomedical Sciences, Cedars-Sinai Medical Center, Los Angeles, California, USA.; 8 Cellular and Molecular Biology Program, University of Michigan, Ann Arbor, Michigan, USA

## Abstract

Prior infection elicits durable reprogramming in myeloid cells and their progenitors; however, the long-term consequences of this reprogramming are not well understood. We previously established a murine model of sepsis survival induced by cecal ligation and puncture which results in enhanced lung injury responses to lipopolysaccharide. In this model, classical monocytes from post-sepsis mice display persistently enhanced cytokine expression after lipopolysaccharide. To test the hypothesis that inflammatory reprogramming of monocytes mediates enhanced lung injury in post-sepsis mice, depletion and/or adoptive transfer was performed three weeks and three months after sepsis. Transcriptomic and epigenomic pathways associated with monocyte reprogramming and shifts in novel monocyte subsets were determined after sepsis in mice and humans. Monocytes from post-sepsis mice mediated enhanced LPS-induced lung injury and promoted neutrophil degranulation. Prior sepsis enhanced JAK-STAT signaling and AP-1 binding in monocytes, shifting toward the neutrophil-like monocyte subset and their progenitors. Similar neutrophil-like monocyte shifts were observed in adult sepsis patients and monocyte counts were predictive of 90-day mortality. We conclude that sepsis induces inflammatory memory affecting bone marrow progenitors and monocyte subsets predisposing to lung injury. These observations serve as a foundation for future investigations on neutrophil-like monocytes and inflammatory program interaction in tissue injury responses.

## Summary

Sepsis shifts the immune program and ontogeny of monocytes and their progenitors contributing to long-term risk for new organ injury.

## Introduction:

Infection can elicit durable reprogramming in immune cells and their progenitors leading to enhanced (i.e., primed or trained) or suppressed (i.e., tolerant) responses to secondary stimuli^1–5^. This reprogramming occurs via changes in transcriptional, epigenetic, and metabolic pathways which may persist for weeks to months^3,6–11^. The mechanisms through which cells are reprogrammed and the contexts in which immune reprogramming promotes beneficial (e.g., host defense) or detrimental (e.g., tissue injury) responses to secondary insults are vastly underexplored.

Sepsis is a leading cause of infection-related hospitalization^12^. Nearly 40 million people survive sepsis each year^13^. Half of these patients are rehospitalized or die in the subsequent year^14–18^, many with new organ injury. For example, 1 in 20 patients with sepsis are rehospitalized with new injurious lung conditions, including aspiration pneumonitis, exacerbation of chronic respiratory disease, and respiratory failure^14,16^. Patients with elevated inflammatory markers (e.g., circulating IL-6, CRP, or leukocytes) at hospital discharge are at particularly increased risk for rehospitalization and death^19–22^. As such, understanding immune trajectories after sepsis is a research priority^23^. The extent to which respiratory complications are attributable to persistent inflammation/immune reprogramming, the specific cells that are reprogrammed, and the mechanisms through which immune reprogramming predisposes to lung injury in sepsis survivors are not known.

To determine the role of immune reprogramming on lung injury in sepsis survivors, we previously developed a model of sterile lung injury in mice that have survived sepsis. In this model, cecal ligation and puncture (CLP) predisposes to enhanced lung injury in response to intranasal (i.n.) lipopolysaccharide (LPS) administered 3 weeks later^24^. Post-CLP mice also experience persistent monocytosis and enhanced *Tnf* expression in classical (Ly6C^hi^) monocytes following LPS administration^24^. Taken together, these data suggest that persistently primed monocytes induced by CLP predispose to enhanced lung injury.

During acute lung injury, Ly6C^hi^ monocytes recruited from the bone marrow (BM) can either promote or mitigate alveolar barrier dysfunction, suggesting diverse monocyte functional states^25^. Heterogeneity in monocyte function may depend on the inflammatory stimulus; in gram-negative bacterial infection, monocytes are protective, whereas in influenza infection, they are detrimental^26,27^. The developmental origins of monocytes in the BM may also determine their function^28^. For example, Ly6C^hi^ monocyte subsets with different functional capacities (e.g., phagocytosis, cytokine secretion, chemotaxis) are encoded epigenetically at the progenitor level, and functionality is maintained through BM transplantation and infection^29,30^. Distinct BM progenitors also produce specific Ly6C^hi^ monocyte subsets (e.g., neutrophil-like and dendritic cell-like) identified by their transcriptional signatures^31–34^; however, their functional roles and relationship with progenitor-derived functions remain unclear. These data imply monocyte progenitor composition determines the recruitable monocyte subsets and therefore functional outcome in response to a stimulus. However, superimposed on progenitor-derived functional heterogeneity, immune reprogramming by prior infection can further alter monocyte function through durable epigenetic changes (i.e., trained immunity) in BM progenitors and mature cells, leading to either protective or detrimental immune responses to secondary immune challenge^35–44^. The extent to which monocyte hematopoietic origins and inflammatory program interact to regulate functional heterogeneity in acute lung injury is poorly understood.

Here, we address fundamental questions in functional heterogeneity and reprogramming of monocytes including: 1) whether monocytes enhance lung injury after sepsis, 2) the durability of this effect, and 3) the underlying mechanisms. We hypothesized that Ly6C^hi^ monocytes are primed by prior sepsis and mediate enhanced lung injury in post-CLP mice. Antibody-mediated depletion and/or adoptive transfer of Ly6C^hi^ monocytes in three week (3-wk) and three month (3-mo) post-CLP mice demonstrated that prior CLP persistently alters Ly6C^hi^ monocytes to enhance lung injury and promote neutrophil degranulation. Evaluation of durable functional, transcriptional, and epigenetic reprogramming of Ly6C^hi^ BM monocytes revealed a shift in monocyte and progenitor subsets and monocyte inflammatory program after CLP. Finally, we demonstrated similar shifts in monocyte subsets in patients with acute infections and that monocyte counts are predictive of poor outcomes in patients who survive sepsis.

## Results

### Depletion of Ly6C^hi^ monocytes from post-CLP mice attenuates the lung injury response to LPS

**Figure 1.**
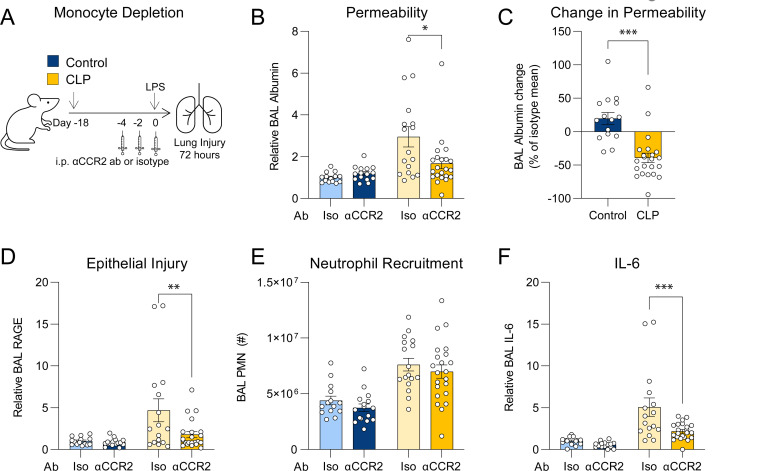
Monocyte depletion improves lung injury and inflammation in post-CLP mice. (A) Experimental design for monocyte depletion an anti-CCR2 antibody (αCCR2) or isotype (Iso) prior to i.n. LPS. Lung permeability assessed by BAL albumin (B), change in permeability relative to isotype-treated for each condition (C), epithelial injury assessed by BAL RAGE (D), neutrophil recruitment (E), and BAL IL-6 (F) shown 72 hours after i.n. LPS. n = 4–8 per group, 3 cohorts; 2 of 18 mice in the iso-treated CLP group died by 72 hours, no other deaths were recorded. BAL protein levels expressed relative to isotype-treated unoperated control mice (light blue bar). Mean ± SEM, Sidak post-hoc p-value (B, D-F), Welch’s t-test (C) shown. * p < 0.05, ** p < 0.01, *** p < 0.001. CLP, Cecal Ligation and Puncture. LPS, Lipopolysaccharide. BAL, bronchoalveolar lavage. RAGE, receptor for advanced glycation end-products. PMN, polymorphonuclear.

To determine whether Ly6C^hi^ monocytes are required for the enhanced lung injury in 3-wk post-CLP mice^24^, we depleted monocytes via anti-CCR2 antibody (αCCR2) administered before i.n. LPS exposure (Figure 1A) . Using flow cytometry (Figure S1), we confirmed that αCCR2 treatment depleted monocytes and monocyte-derived exudate macrophages (Figure S2).

Post-CLP mice treated with αCCR2 antibody (post-CLP_αCCR2_) had significantly reduced BAL albumin relative to post-CLP_ISO_ mice 72 hours after LPS (Figure 1B), indicating decreased lung permeability. Importantly, monocyte depletion in naïve mice enhanced lung injury, as previously reported^26^, whereas monocyte depletion after CLP attenuated lung injury (Figure 1C). Accordingly, there was less alveolar epithelial injury^45^, as measured by BAL RAGE, in post-CLP_αCCR2_ relative to post-CLP_ISO_ mice (Figure 1D). However, neutrophil recruitment to the airspaces was unchanged (Figure 1E). Monocyte depletion significantly reduced BAL IL-6 (Figure 1G) but had little effect on other inflammatory mediators (Figure S3A–C). Taken together, these data confirm that monocytes mediate enhanced lung injury in post-CLP mice following LPS, but not in control mice, suggesting that CLP reprograms monocytes from a protective phenotype toward an injurious phenotype.

### Adoptive transfer of Ly6C^hi^ monocytes from post-CLP mice enhances LPS-induced lung injury

**Figure 2.**
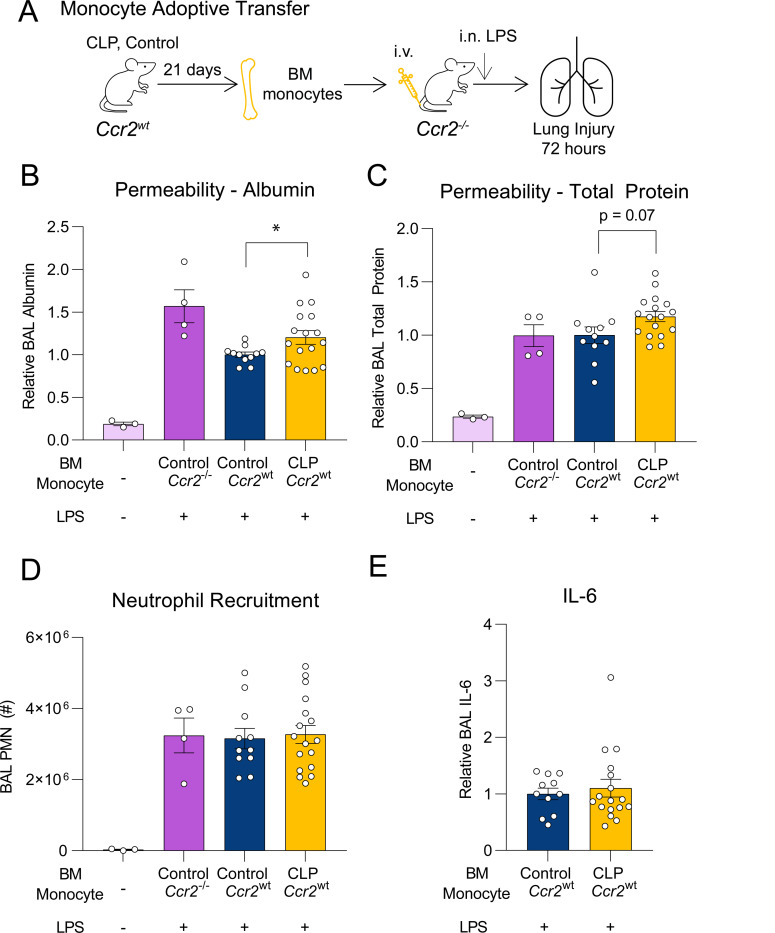
Adoptive transfer of post-CLP monocytes enhances the lung injury response to LPS. (A) Bone marrow Ly6C^hi^ monocytes from *Ccr2*^−/−^, *Ccr2*^*wt*^ age-matched unoperated control, and *Ccr2*^*wt*^ 3-wk post-CLP mice were isolated and administered into *Ccr2*^−/−^ i.v. with concurrently with i.n. LPS. Alveolar permeability assessed by BAL albumin or total protein (B, C), neutrophil recruitment (D), and BAL IL-6 (E) shown 72 hours after i.n. LPS. BAL protein measurements expressed relative to unoperated control *Ccr2*^*wt*^ transfer for each cohort. n = 3–6 per group, 3 cohorts. Mean ± SEM, Welch’s t-test p-value shown. * p < 0.05, ** p < 0.01. CLP, Cecal Ligation and Puncture. LPS, Lipopolysaccharide. BAL, bronchoalveolar lavage. PMN, polymorphonuclear.

To determine whether 3-wk post-CLP monocytes are sufficient to enhance the lung injury response to LPS, we adoptively transferred Ly6C^hi^ monocytes from the BM of post-CLP and unoperated control mice to *Ccr2*^−/−^ mice, followed by i.n. LPS administration (Figure 2A). This approach isolates the effect of the adoptively transferred *Ccr2*^*wt*^ monocytes from the confounding effect of endogenously recruited monocytes in the recipient mice.

Consistent with prior literature^26^, transfer of *Ccr2*^*wt*^ unoperated control monocytes reduced lung permeability, measure by BAL albumin, as compared to transfer of *Ccr2*^−/−^ monocytes (Figure 2B). Post-CLP monocyte transfer resulted in increased BAL albumin and total protein relative to *Ccr2*^*wt*^ unoperated control monocytes (Figure 2B,C). There were no differences in neutrophil recruitment to the airspace (Figure 2D) or levels of most cytokines and chemokines (Figure 2E, Figure S4). Collectively, we show that post-CLP Ly6C^hi^ monocytes are detrimental in LPS-induced lung injury in contrast to the more protective functional program of unoperated control Ly6C^hi^ monocytes.

### Post-CLP Ly6C^hi^ monocytes promote neutrophil S100A8/A9 release and degranulation in response to LPS

**Figure 3.**
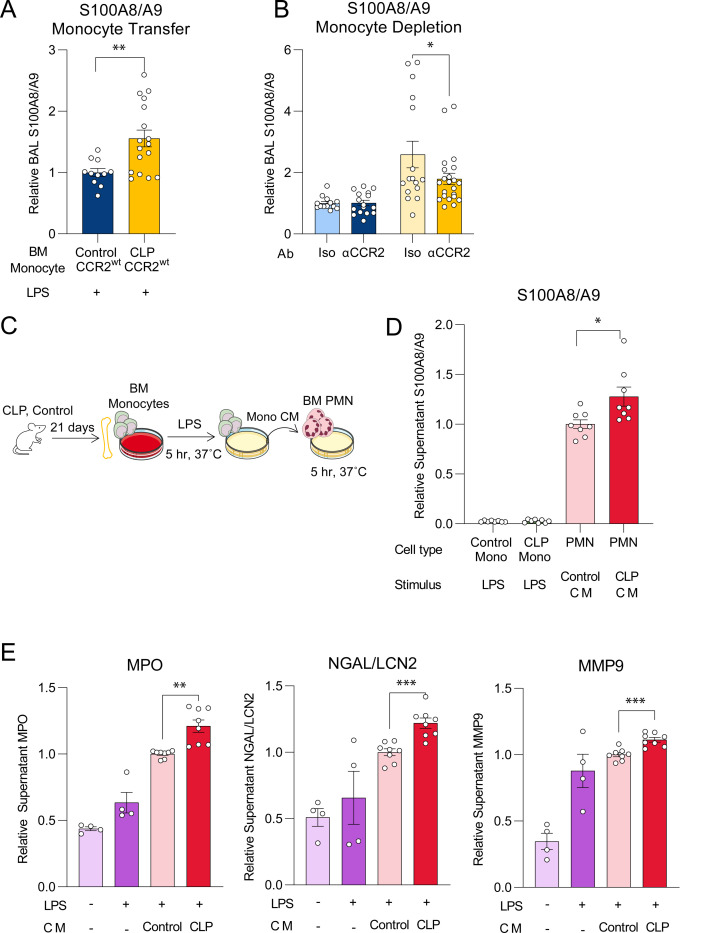
Post-CLP monocytes promote S100A8/A9 release and degranulation of neutrophils. (A) BAL S100A8/A9 following adoptive transfer and (B) antibody-mediated depletion of bone marrow Ly6C^hi^ monocytes. (C) Ex vivo stimulation of neutrophils with monocyte conditioned media. (D) Monocyte and neutrophil S100A8/A9 production in supernatants following stimulation with LPS or monocyte conditioned media (CM), respectively. (E) Myeloperoxidase (MPO), neutrophil gelatinase-associated lipocalin (NGAL/LCN2), matrix metallopeptidase 9 (MMP9) were measured to assess degranulation. n = 4 mice per group, 2 cohorts. Supernatant protein expressed relative to unoperated control monocyte CM. Mean ± SEM, Welch’s t-test p-value shown. * p < 0.05, ** p < 0.01, *** p <0.01. CLP, Cecal Ligation and Puncture. LPS, Lipopolysaccharide. BAL, bronchoalveolar lavage.

Lung permeability in LPS-induced lung injury is dependent on neutrophil recruitment and activation^46–48^. Despite improvement in lung permeability, neutrophil recruitment was similar after monocyte depletion and adoptive transfer. Therefore, we hypothesized that neutrophil activation and degranulation may be differentially regulated by post-CLP monocytes. BAL levels of S100A8/A9, a damage associated-molecular pattern protein released upon neutrophil activation which also promotes degranulation^49–52^, were previously elevated in the BAL of post-CLP mice following i.n. LPS (7). We found that adoptive transfer of post-CLP monocytes increased, whereas monocyte depletion reduced BAL S100A8/A9 in post-CLP mice (Figure 3A,B), suggesting monocyte regulation of neutrophil activation and degranulation.

To test whether post-CLP monocytes directly induce degranulation in neutrophils, we isolated BM Ly6C^hi^ monocytes from post-CLP mice, stimulated with LPS, and transferred the monocyte conditioned media (CM) to naïve BM neutrophils (Figure 3C). Post-CLP monocyte CM induced more release of S100A8/A9 (Figure 3D) and other neutrophil granule proteins (MPO, NGAL, MMP9) than unoperated control monocyte CM (Figure 3E). These data suggest that post-CLP monocytes enhance neutrophil activation and degranulation contributing to enhanced lung injury.

### Post-CLP Ly6C^hi^ monocytes show a pro-inflammatory transcriptional and epigenetic activation state characterized by enrichment of TLR4, HIF-1α, JAK-STAT signaling and AP-1 binding

These data imply that sepsis reprograms Ly6C^hi^ monocytes toward a primed pro-inflammatory state. Immune priming following a primary stimulus is attributable to activated transcription and epigenetic alterations that promote more robust immune responses to secondary stimuli^2^. We hypothesized that CLP would induce persistent alterations in the transcriptome and/or epigenome of monocytes. To determine the transcriptional and epigenetic alterations mediating monocyte priming, we performed bulk RNA- and ATAC-sequencing on mature Ly6C^hi^ monocytes (Figure S5).

**Figure 4.**
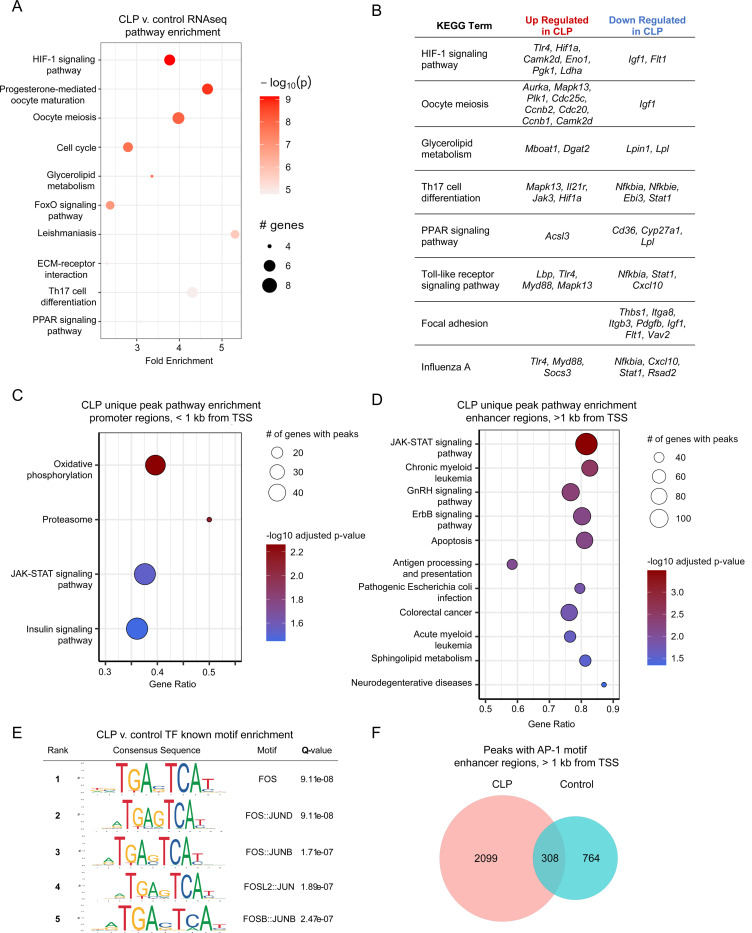
Transcriptional and epigenetic pathways contributing to post-CLP monocyte priming. Ly6C^hi^ monocytes were isolated from the bone marrow and sorted (Lin-/Ly6G-/CD115+/CD11bhi/Ly6Chi/c-Kit-/Flt3-/) differential expression and pathway enrichment performed. (A) Top 10 transcriptomic pathways enriched in 3-wk post-CLP monocytes relative to control. (B) Selected transcriptomic pathways enriched in post-CLP monocytes with upregulated and downregulated genes per pathway. (C, D) In a separate experiment, ATAC-seq was performed, unique peaks were identified, and pathway enrichment performed. (E) Known motif enrichment performed using control monocytes as the genomic background. (F) Quantification of peaks containing the AP-1 binding motif in post-CLP and control monocytes. n = 4–5 mice per group. CLP, Cecal Ligation and Puncture. TSS, transcription start site.

Pathway analysis of gene expression data revealed that post-CLP monocytes had enriched HIF-1α signaling, cell cycle, glucose/lipid metabolism, and inflammatory signaling pathways relative to control (Figure 4A). They exhibited upregulation of TLR4 pathway genes (*Tlr4*, *Lbp, Myd88*, *Mapk13*) and downregulation of TLR4 pathway inhibitors (*Nfkbia, Nfkbie*) (Figure 4B). There was also upregulation of JAK-STAT pathway genes (*Jak3*) and downregulation of some anti-inflammatory genes (*Igf1*). ATACseq revealed enrichment of uniquely accessible promoter regions associated with genes in glycolytic and inflammatory pathways in post-CLP mice (Figure 4C). JAK-STAT signaling was enriched in both the promoter (Figure 4C) and enhancer (Figure 4D) regions in post-CLP monocytes. In contrast, control monocyte promoter regions were enriched for pathways homeostatic including vesicle transport, fatty acid biosynthesis/signaling, acetyl-CoA handling, and nucleotide metabolism (Figure S6).

To identify the transcription factors associated with open genomic regions, which may indicate active or poised transcription, we performed known motif enrichment in post-CLP monocytes relative to control monocytes^53^. This identified FOS:JUN (AP-1) motifs in enhancer regions of post-CLP monocytes (Figure 4E), including a nearly 3-fold increase in the number of genes with at least one peak containing the AP-1 motif (Figure 4F). Together these data support a shift in BM Ly6C^hi^ monocyte transcriptional and epigenetic activation in post-CLP mice toward a persistent pro-inflammatory state.

### CLP induces a shift in ontogeny toward neutrophil-like (NeuMo) Ly6C^hi^ monocytes and their progenitors

**Figure 5.**
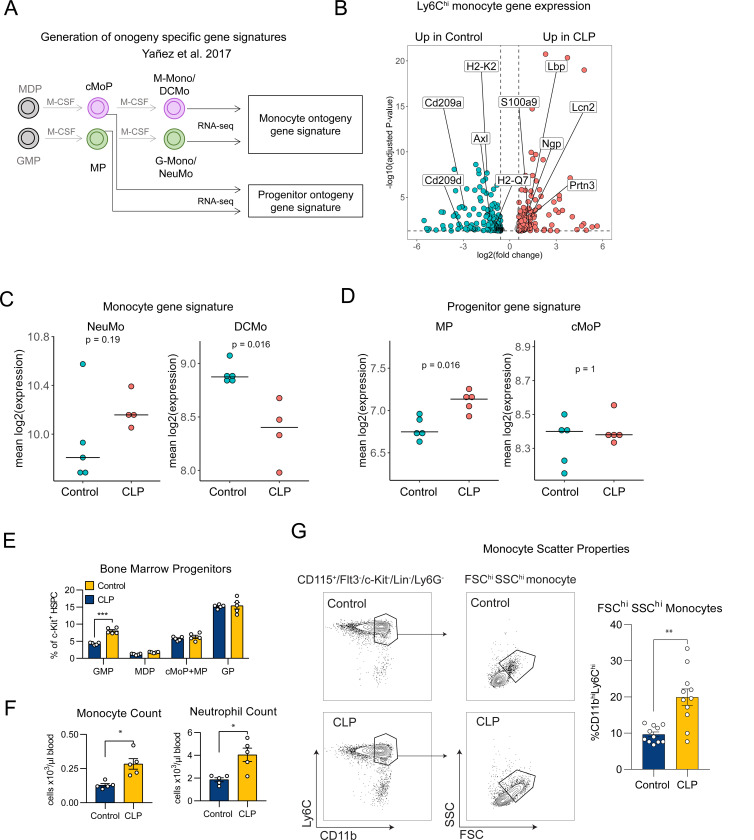
Neutrophil-like monocytes and their progenitors are expanded in post-CLP mice. Ly6C^hi^ monocytes (Lin^−^/Ly6G^−^/CD115^+^/c-Kit^−^/Flt3^−^/CD11b^hi^/Ly6C^hi^) and BM progenitors (MP+cMoP enriched, Lin^−^/Ly6G^−^/CD115^+^/Flt3^−^/c-Kit^+^) were isolated from the bone marrow for RNAseq, and differential expression analysis was performed. (A) Gene sets for GMP- and MDP-derived monocyte-committed progenitors (MP and cMoP, respectively) and Ly6C^hi^ monocytes (NeuMo and DCMo, respectively) were defined in a prior ex vivo experiment ^31^. (B) Select genes in Ly6C^hi^ monocytes consistent with upregulation of a neutrophil-like signature and downregulation of a DC-like signature are indicated in post-CLP monocytes relative to unoperated control. Gene set enrichment for monocyte subsets (C) and their progenitors (D) in control and post-CLP monocytes. (E) Quantification of myeloid progenitors by flow cytometry. (F) Peripheral blood counts for monocytes and neutrophils. (G) Side and Forward scatter properties of Ly6Chi monocytes in post-CLP and unoperated control. n = 4–5 per group, 1 cohort (A-F), n= 4–5 per group, 2 cohorts (G). Mean, Wilcoxon rank-sum (C, D), Mean ± SEM and Welch’s t-test (E-G) are shown. * p < 0.05, ** p < 0.01, *** p <0.01. MDP, monocyte-dendritic cell progenitor. cMoP, common monocyte progenitor, DCMo, dendritic cell-like monocyte. GMP, granulocyte-monocyte progenitor. MP, monocyte progenitor. NeuMo, neutrophil-like monocyte. CLP, cecal ligation and puncture. GP, granulocyte progenitor.

Subsets of Ly6C^hi^ monocytes with distinct hematopoietic origins (i.e., distinct ontogeny) have been identified^28^. Neutrophil-like (NeuMo) monocyte arise from the granulocyte-monocyte progenitor (GMP) via their own monocyte-committed progenitor (MP), whereas dendritic cell (DC)-like (DCMo) monocytes arise from the monocyte-DC progenitor (MDP) via the common monocyte progenitor (cMoP) (Figure 5A). However, the functional significance of these subsets is unknown, and they have not been characterized in the context of prior sepsis and lung injury. NeuMo and DCMo are identified by transcriptional signatures, as there are no accepted surface markers to distinguish these populations^28,31,34^. We found that post-CLP monocytes upregulated more neutrophil genes (e.g., neutrophil granule protein, *Ngp*, proteinase-3 *Prtn3*, lipocalin-2 *Lcn2*) and downregulated dendritic cell specific genes (e.g., dendritic cell-specific intercellular adhesion molecule-3-grabbing non-integrin (DC-SIGN), *Cd209a*) (Figure 5B). Therefore, we hypothesized that CLP induces a shift in monocyte ontogeny with expansion of the NeuMo subset.

To assess this, we performed gene signature enrichment analysis for NeuMo and DCMo in the Ly6C^hi^ monocyte fraction using subset-specific NeuMo and DCMo gene lists^31^. Monocytes from post-CLP mice were enriched for the NeuMo signature, while control monocytes were enriched for the DCMo signature (Figure 5C). To further assess NeuMo expansion, we also tested for enrichment of monocyte-committed MP and cMoP gene signatures within a MP+cMoP enriched fraction (Figure S5). Confirming our hypothesis, post-CLP BM progenitors were enriched for an MP signature (Figure 5D). Given that NeuMo are derived from the GMP, we hypothesized that GMP would be expanded in the BM of post-CLP mice. Using established surface markers (Figure S7)^54^, we found increased GMP progenitors in post-CLP mice relative to control, without changes in MDP (Figure 5E). Consequently, we observed increased monocytes and neutrophils in peripheral blood (Figure 5F). To independently validate these findings, we analyzed forward-scatter (FSC) and side-scatter (SSC) properties of Ly6C^hi^ monocytes since NeuMo are larger and more granular ^31^. We found two populations of monocytes within the Ly6C^hi^ fraction: FSC^lo^SSC^lo^ and FSC^hi^SSC^hi^. Post-CLP mice exhibited a 2–3 fold increase in FSC^hi^SSC^hi^ Ly6C^hi^ cells, consistent with NeuMo expansion. Taken together, we conclude that sepsis induced by CLP is associated with a shift in monocyte ontogeny toward the GMP pathway with enrichment of MP and NeuMo populations. Given the role of post-CLP monocytes in enhancing LPS-induced lung injury (Figure 1,2), enrichment of the NeuMo subset may promote enhanced lung injury in post-CLP mice.

### Enhanced lung injury in post-CLP mice persists for up to 3 months

**Figure 6.**
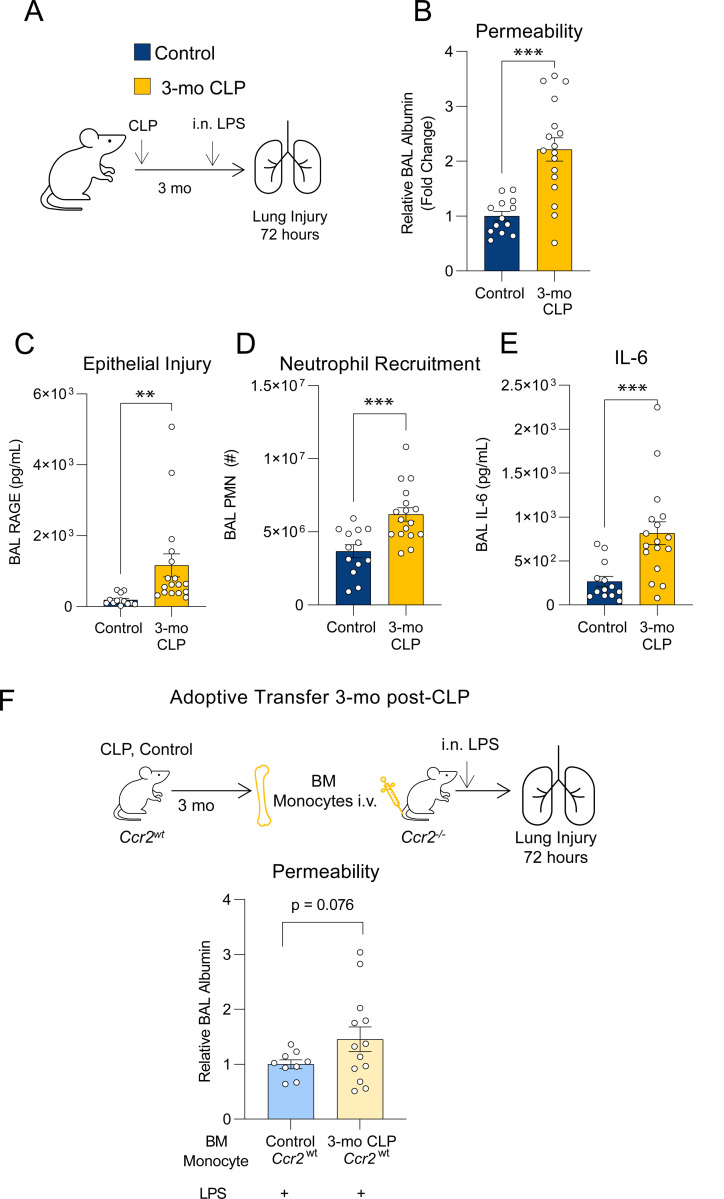
Monocyte mediated enhanced lung injury in post-CLP mice is durable. (A) 3-mo post-CLP and age-matched control mice underwent i.n. LPS administration. Alveolar permeability assessed by BAL albumin (B), epithelial injury assessed by BAL RAGE, neutrophil recruitment (D), and BAL IL-6 (E) measured 72-hr after i.n. LPS. (F) Bone marrow Ly6C^hi^ monocytes were isolated from *Ccr2*^*wt*^ age-matched unoperated control, and *Ccr2*^*wt*^ 3-mo post-CLP mice and administered into *Ccr2*^−/−^ mice i.v. concurrently with i.n. LPS, alveolar permeability assessed by BAL albumin. n = 5–10 per group, 2 cohorts (A-E), n = 2–6 per group, 3 cohorts (F). Mean ± SEM, Welch’s t-test p-value shown. * p < 0.05, ** p < 0.01, *** p < 0.001. CLP, Cecal Ligation and Puncture. LPS, Lipopolysaccharide. BAL, bronchoalveolar lavage. RAGE, receptor for advanced glycation end-products. PMN, polymorphonuclear. BM, bone marrow.

Since immune reprogramming at the progenitor level can lead to durably enhanced inflammatory responses^22,35,44^, we hypothesized that the predisposition to enhanced lung injury seen in post-CLP mice would persist beyond three weeks. We evaluated the lung injury response at 3 months (3-mo) post-CLP (Figure 6A) and observed significantly increased lung permeability and epithelial injury compared to control mice (Figure 6B, 6C). This was associated with increased airspace neutrophils and IL-6 (Figure 6D, 6E). Adoptive transfer of monocytes from 3-mo post-CLP mice resulted in increased lung permeability relative to transfer of unoperated control monocytes (Figure 6F). These data confirm durably enhanced lung injury following recovery from sepsis which is mediated by BM monocytes. Given the half-life of monocytes is hours to days^55,56^, the persistence of this phenotype implies functional reprogramming of monocytes at the progenitor level.

### Neutrophil-like gene signature is enriched in patients with acute sepsis

Given the expansion and apparent functional implications of NeuMo in murine sepsis survival, we sought to establish whether monocytes with a neutrophil-like gene signature are similarly mobilized/expanded in human patients with sepsis. To explore this, we took advantage of publicly available bulk and single cell RNA sequencing (scRNAseq) datasets for human CD14+ monocytes^57–59^. To assess enrichment of monocyte subsets, we utilized previously identified human neutrophil-like and DC-like gene signatures with conserved transcription between mice and humans^60^.

**Figure 7.**
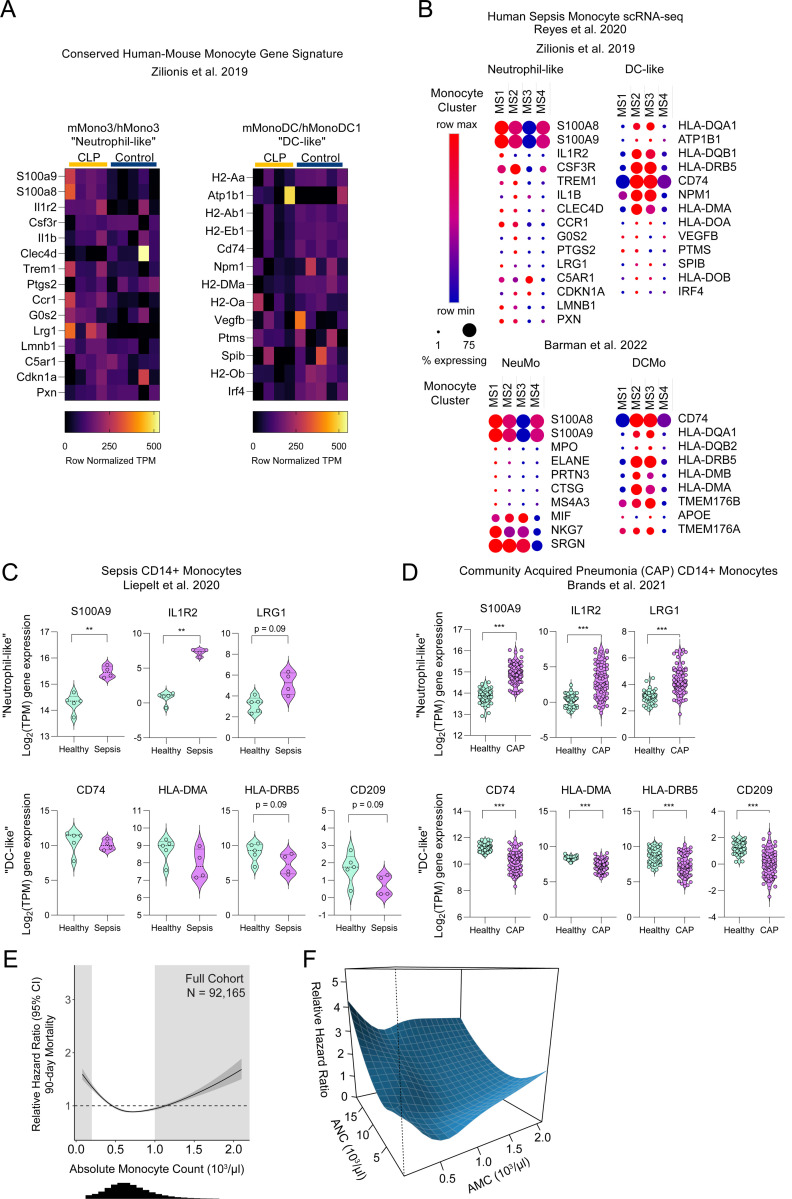
Neutrophil-like monocyte transcriptional signature is enriched in acute infection and blood monocyte counts are predictive of long-term outcomes in adult sepsis survivors. (A) Heatmap showing gene lists for conserved human-mouse neutrophil-like and DC-like subsets in 3-wk post-CLP and control mice ^60^. (B) Bubble plots showing the enrichment of conserved human-mouse neutrophil-like or DC-like monocyte ^60^ or mouse-specific NeuMo and DCmo ^34^ genes across monocyte clusters in scRNA-seq analysis from patients with acute bacterial infection ^57^. (C,D) Violin plots of neutrophil-like and DC-like gene expression in CD14+ monocytes in patients with acute sepsis (n = 4 sepsis, 5 healthy) ^58^, or community acquired pneumonia (n = 69 CAP, 41 healthy) ^59^. (E) Unadjusted Cox proportional hazard regression (95% CI, dark gray) with relative hazard ratio for 90-day mortality for absolute monocyte count (AMC), histogram of AMC distribution shown below. Light gray boxes indicate values outside of the normal clinical range. (F) Three-dimensional interaction surface showing the relationship of AMC (x-axis) and absolute neutrophil count (ANC, z-axis). The y-axis shows the effect of the combination of parameters on relative hazard ratio for 90-day mortality. Mean, 25^th^ and 75^th^ percentile shown (C, D). * p < 0.05, ** p < 0.01, *** p < 0.001. CLP, cecal ligation and puncture. NeuMo, mouse neutrophil-like monocyte. DCMo, mouse dendritic cell-like monocyte.

We first evaluated this conserved human-mouse signature in our post-CLP and control monocytes and found upregulation of neutrophil-like genes and downregulation of DC-like genes in post-CLP monocytes relative to control (Figure 7A). We next evaluated enrichment of these subsets across monocyte sub-clusters (MS1-MS4) identified by scRNAseq in patients with acute bacterial infection and sepsis, hypothesizing that one cluster may represent the neutrophil-like population^57^. Cluster MS1 had the most consistent upregulation of the mouse-human conserved neutrophil-like gene signature and downregulation of the DC-like gene signature, suggesting a more neutrophil-like and less DC-like state (Figure 7B). Using mouse-specific NeuMo and DCMo gene signatures^34^, a similar pattern was observed (Figure 7B). To validate these findings, we evaluated individual neutrophil-like and DC-like genes from bulk RNAseq of CD14+ monocytes from patients with sepsis or pneumonia ^58,59^. We found upregulation of the conserved neutrophil-like genes (*S100A9*, *IL1R2*, *LRG1*) and downregulation of DC-like genes (*CD74*, *HLA-DMA*, *HLA-DRB5*), as well as the mouse DCMo gene *CD209* (Figure 7C, 7D). These data suggest monocyte ontogeny is shifted toward neutrophil-like GMP-derived monocytes in both acute sepsis and pneumonia.

### Monocyte count in patients surviving sepsis is a predictor of long-term risk for 90-day mortality

Patients who survive sepsis are at increased risk of long-term rehospitalization and death^15,61^. Given that monocytes mediate acute lung injury in sepsis survivor mice (Figure 1, 2) and patients with acute sepsis mobilize similar monocyte subsets, we hypothesized that mobilization of monocytes may be associated with long-term outcomes after sepsis in humans. Since we previously showed that an abnormal leukocyte count at the time of hospital discharge predicts 90-day rehospitalization and/or death^21^, we hypothesized that absolute monocyte count (AMC) measured at hospital discharge would also predict 90-day mortality in patients surviving sepsis.

Using a United States Veteran’s Affairs (VA) health system dataset^62^, we identified 92,165 hospitalizations that met inclusion criteria (Figure S8). Consistent with our prior results^21^, 13.6% of live discharges in the cohort were associated with a death within 90-days (Supplemental Table 1). Patients with 90-day mortality were older (median (IQR) 72 (66, 83) v. 68 (61,75) years of age) and had more comorbidity (median (IQR) 8 (6, 10) v. 6 (4, 8) Charlson co-morbidities) than 90-day survivors. While the number of acute organ dysfunctions was similar between groups, patients who died within 90 days of discharge had more renal dysfunction (65.6% v. 59.7%), hematologic dysfunction (16.7% v. 9.4%), vasopressor use (9.9% v. 6.6%), hepatic dysfunction (9.9% v. 6.6%), and mechanical ventilation (6.1% v. 3.9%); and more commonly had ICU admission (37.9% v. 26.1%) and longer hospital length of stay (median IQR, 8 (5,12) v. 6 (4, 9) days).

In bivariate analysis, absolute monocyte count (AMC) was associated with an increased hazard for 90-day mortality (Figure 7E). The biphasic distribution of increased hazard ratio for both low and high AMC was consistent with our prior observations in other leukocyte parameters^21^. Since absolute neutrophil count (ANC) is associated with 90-day mortality after sepsis^21^ and neutrophil/monocyte mobilization occur concurrently in our murine model (Figure 5), we analyzed whether the predictive value of monocyte count was dependent on neutrophil count by plotting the hazard ratio surface for AMC and ANC for 90-day mortality (Figure 7F). The hazard ratio for AMC increased most dramatically as ANC increased. In conclusion, AMC measured at hospital discharge is predictive of long-term outcomes in patients who survive hospitalization for sepsis and the predictive value of AMC is highly dependent on the concomitant ANC.

## Discussion:

Herein, we demonstrated that sepsis induced by CLP durably reprograms Ly6C^hi^ monocytes to a primed inflammatory state which predisposes to enhanced LPS-induced lung injury. Ly6C^hi^ monocyte subsets following CLP are shifted toward NeuMo and their progenitors (MP and GMP), suggesting that specific Ly6C^hi^ monocyte subsets of distinct BM origins govern the trajectory of lung injury. We identified several transcriptomic and epigenomic pathways associated with enhanced lung injury after CLP, including enrichment of TLR4 and JAK-STAT signaling. We validated our findings in humans, showing that monocytes in infected patients are enriched for a neutrophil-like/NeuMo transcriptional signature and that monocyte count at hospital discharge for sepsis is predictive of long-term clinical outcomes. These data support a conceptual model whereby by sepsis shifts the immune program and ontogeny of monocytes and their progenitors and contributes to long-term risk for new organ injury.

Though inflammatory reprogramming during acute sepsis is associated with poor clinical outcomes^5^, less is known about durable reprogramming after sepsis and its role in mediating long-term outcomes, including new organ injury. In this study, CLP primed Ly6C^hi^ monocytes to facilitate sterile lung injury and inflammation in response to LPS. Sepsis survival was also associated with shift in bone marrow myeloid progenitors in mice and persistent myelopoiesis in mice and humans. These findings are consistent with prior investigations of inflammatory reprogramming through innate immune memory, whereby various insults reprogram hematopoietic stem cells (HSC) leading to enhanced responses to secondary stimuli and a myeloid differentiation bias^35,38,44^. This reprogramming may protect the host from secondary bacterial infections^38,40,44^, however, it can also be maladaptive – enhancing secondary collagen induced arthritis^41^ and endotoxic shock^38^ in mice. The mechanisms of maladaptive reprogramming are not well described. Here we show that post-CLP monocytes enhance neutrophil degranulation, suggesting that maladaptive reprogramming may promote tissue injury through augmenting monocyte-neutrophil crosstalk. As neutrophil degranulation is a primary mediator of barrier tissue injury and a key pathogenic mechanism in the development of lung injury and acute respiratory distress syndrome (ARDS)^63,64^, maladaptive monocyte programming may represent an important mediator of the development and progression of acute lung injury syndromes in patients with or without prior sepsis.

An emerging literature has revealed that Ly6C^hi^ monocytes are composed of heterogenous subpopulations^31–34^. The biological contexts in which monocyte subsets arise and their functional role in disease pathogenesis are poorly understood^28,65^. Prior to this work, Ly6C^hi^ monocyte subsets (NeuMo and DCMo) had been identified in homeostasis and sterile inflammation^31–34^. To our knowledge, we have observed for the first time that NeuMo are induced by bacterial sepsis in mice and humans. Interestingly, in humans we identified the MS1 sub-cluster as being most “neutrophil-like” (Figure 7B), and the MS1 transcriptional signature was previously found to be predictive of bacterial infection^57^. Our data also imply a functional role for NeuMo in enhancing LPS-induced acute lung injury; concordantly, patients with upregulated MS1 transcriptional signature appear to be at increased risk for ARDS^66^. In contrast, others have shown that Ym1^+^ (chitinase-like 3, *Chil3*) NeuMo are protective against experimental colitis^32,67^. Similarly, during emergency monopoiesis monocytes bearing a neutrophil-like signature, including *Ym1*, were found to be protective against brain injury^68^. In light of these prior data, our results suggest that 1) post-CLP NeuMo represent an alternate inflammatory program without the protective features of Ym1^*+*^ Ly6C^hi^ NeuMo and/or 2) the role of NeuMo in inflammatory organ injury is disease or tissue-dependent. These results represent a paradigm shift in our understanding of monocytes in acute lung injury: that functional heterogeneity may be determined by the monocyte subset and/or progenitor pool present prior to injury. Therefore, studying monocyte subset variation may be beneficial for predicting the risk for development of organ injury including new sepsis or lung injury.

Innate immune memory is a persistent inflammatory state that occurs when a primary inflammatory exposure leads to enhanced inflammatory responses to various secondary stimuli for weeks to months^35–43^.This occurs through opening of chromatin, binding of transcription factors, and modification of histones by a primary stimulus which persist in stem/progenitor cells and facilitate enhanced transcription upon a secondary stimulus^4^. We found enhanced chromatin accessibility, enrichment of the JAK-STAT signaling pathway, and increased Fos:Jun (AP-1) binding motifs in the 3-wk post-CLP monocyte genome, suggesting that a maladaptive innate immune memory phenotype is associated with enhanced lung injury. These findings are supported by prior work that has established JAK-STAT signaling and subsequent induction of AP-1 binding as key to initiating the epigenetic predisposition to inflammation in innate immune memory^22,35,69^. We also found upregulation of both HIF-1α and TLR signaling pathways in post-CLP monocytes 3-wks after CLP which have also been associated with the establishment of innate immune memory^44,70^. Taken with our prior work showing persistent inflammation weeks after CLP^24,71,72^ and persistently enhanced lung injury 3-mos after CLP (Figure 6), it is possible that the 3-wk post-CLP phenotype represents an intermediate stage in the development of immune memory. Importantly, we also see transcriptional changes in the Ly6C^hi^ monocyte pool consistent with shifts in monocyte subsets. It is unclear if these epigenetic changes relate to specific monocyte subsets (i.e., NeuMo) or are broadly experienced by the entire monocyte pool. Trained immunity induced by BCG vaccination in humans is associated with shifts in monocyte subsets, including one subset with increased neutrophil gene expression^69^, suggesting that neutrophil-like monocyte expansion may be a feature of innate immune memory initiation. Future work will delineate how the induction of immune memory/reprogramming across monocyte subsets affects their relationship with beneficial or detrimental responses to secondary stimuli.

There are several limitations to this study. We solely utilized LPS to induce lung injury, future studies will examine how post-sepsis monocyte reprogramming alters host defense and lung injury in the setting of bacterial or viral lung infection. We are currently unable to confirm the relationship of monocyte subsets in human sepsis survivors with long-term clinical outcomes. However, we establish that neutrophil-like monocytes gene expression is increased in multiple cohorts of patients with acute infection and that elevated monocyte counts predict clinical outcomes. Future studies will need to evaluate specific monocyte subsets in the circulation and progenitor populations in the bone marrow to completely understand the long-term inflammatory consequences of sepsis in humans. We attribute enhanced lung injury to a shift in monocyte ontogeny in post-CLP mice based on enrichment of a NeuMo gene signature, future studies using scRNAseq, ontogeny specific reporters, and methods to target specific subsets in health and disease are necessary.

Our findings provide insight into fundamental questions in the emerging field of monocyte heterogeneity^73^: the extent to which monocyte ontogeny and inflammatory context regulate functional heterogeneity in health or disease. In our mouse model of sepsis survival, Ly6C^hi^ monocytes enhance the lung injury response to LPS and promote neutrophil degranulation. We observe for the first time, to our knowledge, that neutrophil-like monocytes are mobilized by sepsis in mice and humans. We also identify a functional role for neutrophil-like monocytes in the pathogenesis of acute lung injury. With the discovery of a potentially ontogeny-specific phenotype, these observations serve as a foundation for future investigations to address key gaps in our understanding of how monocyte subset and inflammatory program might collaborate to enhance tissue injury responses. Finally, the persistent nature of hematopoietic reprogramming after sepsis in mice identifies a potential target for investigation in the prevention of post-sepsis organ injury in humans.

## Materials and Methods

### Mouse studies

Male 8–12 weeks old C57BL/6J (strain 000664) and *Ccr2*^−/−^ (strain 004999) mice were purchased from Jackson Laboratories. All procedures involving animals were undertaken in strict accordance with the recommendations of the Guide for the Care and Use of Laboratory Animals by the National Institutes of Health. The University of Michigan facility maintains a Specific Pathogen Free barrier environment. The study protocol was approved by the University Committee on the Use and Care of Animals of the University of Michigan (protocol number 00008999 and 00010712).

### Sepsis patient cohort study

The study was reviewed by the Veterans Affairs Ann Arbor institutional review boards and was deemed exempt from the need for consent under 45 CFR §46, category 4 (secondary use of identifiable data).

### Cecal ligation and puncture

CLP was performed as previously described^24,72^. Briefly, animal suffering and distress were minimized using analgesia with local lidocaine, as well as anesthesia with ketamine and xylazine. Under aseptic conditions, a 1–2 cm laparotomy was performed. The cecum was ligated with a silk suture and punctured once through-and-through with a 19-gauge needle. The incision was closed with surgical clips. Imipenem/cilastatin (Merck, 0.5 mg/mouse in 200 μl of normal saline) and normal saline (0.5 mL) were administered subcutaneously to all CLP mice immediately following surgery. This method induces polymicrobial bacterial peritonitis with disseminated infection, an average mortality of 10%, and clearance of bacterial cultures within 5–7 days^24,72,74^. By three weeks, there is no difference in weight between groups ^24^. We also did not observe any additional mortality during the three week to three month recovery period, and aerobic peritoneal cultures in 3-mo post-CLP mice remained negative (data not shown).

### Lipopolysaccharide-induced Lung Injury

*Escherichia coli* LPS (O111:B4, Sigma) was administered i.n. at predetermined time points (18 to 84 days) after CLP or in age-matched unoperated control mice. We utilized unoperated control mice because we previously established that sham surgery and unoperated control mice have similar lung injury responses to LPS at 3 weeks post-surgery^24^. Briefly, mice were anesthetized using ketamine and xylazine. LPS dissolved in sterile normal saline (50 μg, 1 μg/μl) was administered 25 μl per nostril. Mice were euthanized 24 or 72 hours after LPS administration. The University of Michigan policy for humane endpoints was followed.

### Monocyte depletion

Monocyte depletion was performed using an anti-CCR2 antibody (clone MC-21,^75^) or rat IgG2b isotype (clone LTF-2, BioXCel or clone MC-67) administered intraperitoneally (i.p., 40 μg) four days prior (day −4), two days prior (day −2), and at the time of LPS administration (day 0). Treatments were randomized within cage. Mononuclear cell counts were assessed by DiffQuick (Baxter) staining on BAL cytospins. At 72 hours after LPS, post-CLP mice treated with anti-CCR2 antibody had less mononuclear cells in the BAL than isotype treated mice, confirming monocyte depletion. One cohort (out of 4 total depletions) showed elevated mononuclear cell counts in the BAL relative to isotype treated control for all mice, this was inconsistent with monocyte depletion and was excluded from analysis.

### BM isolation and immunomagnetic monocyte isolation

BM was isolated from femurs and tibia of post-CLP mice (days 21 and 84) and age-matched control mice. Briefly, mice were euthanized with ketamine and xylazine. In a biological safety cabinet using sterile technique, hind legs were disarticulated and placed in RPMI with 10% Fetal Bovine Serum (FBS), 1% penicillin/streptomycin (P/S). Bones were cleaned and placed briefly into 100% ethanol. Bones were then cut on both ends and flushed with RPMI with 10% FBS, 1% P/S. using a 27-gauge needle. Marrow was disaggregated and then passed through a 100 μm filter to remove debris. Cells were counted on a hemocytometer using Trypan blue. BM from two hind legs was resuspended in 1 mL PBS supplemented with 1 mM EDTA, 2 % FBS. Monocytes were then isolated using the EasySep Monocyte Isolation Kit (STEMCELL) as per manufacturer recommendations. This method consistently led to isolation of approximately 1–2 × 10^6^ cells with 90–95% Ly6C^hi^ monocytes with no difference in purity between conditions.

### Monocyte adoptive transfer

Monocytes from post-CLP, unoperated, and *Ccr2*^−/−^ control mice were isolated and pooled (n = 2–5 mice). *Ccr2*^−/−^ recipient mice were anesthetized with ketamine and xylazine and 1×10^6^ monocytes were administered in 100–200 μl of PBS via tail vein injection. Mice then immediately received i.n. LPS to induce lung injury. Treatments were randomized within cage.

### Monocyte and neutrophil ex vivo culture

Monocytes were isolated from BM and immunomagnetically enriched as above. Monocytes from individual post-CLP and unoperated control mice were resuspended at 2.5 × 10^5^ cells/mL in 2 mL DMEM with 10% FBS with LPS (1 μg/mL) in a 12-well plate and incubated for 5 hours at 37 °C with 5% CO_2_. Plates were removed and placed on ice, supernatants removed and centrifuged at 450 g for 10 minutes at 4 °C. Cell-free supernatants were stored at −20 °C until use.

Neutrophils were isolated from BM as previously described^76^. Briefly, whole BM was isolated from hind legs, red cells were lysed using ACK lysis buffer for 1 minute in 37 °C, and cells were washed with RPMI with 10% FBS, 1% P/S . A density gradient was created layering 3 mL of each of the following: Histopaque 1119 (Sigma-Aldrich, density 1.119 g/mL), Histopaque 1077 (Sigma-Aldrich, density 1.077 g/mL), and whole BM from two hind legs in ice cold PBS. Centrifugation was performed at 872 g at room temperature without brake for 30 minutes. Neutrophils were collected from the Histopaque interface and washed twice with RMPI with 10% FBS with 1% penicillin/streptomycin. Neutrophils from 6 naïve mice were pooled. Cells were counted by trypan blue exclusion on a hemacytometer and purity was assessed to be >90% using DiffQuick (Baxter) on cytospins. Neutrophils were resuspended at 5 × 10^6^ cells/mL in DMEM with 10% FBS and transferred to wells containing 1 mL of thawed monocyte conditioned media (post-CLP or control) or fresh basal media with or without LPS (1 μg/mL) in a 12-well plate. Cells were incubated for 5 hours at 37 °C with 5% CO_2_. Supernatants were isolated as above.

### Serum collection and complete blood cell counts

Whole blood was obtained by puncture of the right ventricle using a heparinized 1 mL syringe and a 26-gauge needle. Needles were removed, blood ejected, and then placed on ice for no more than 1 hour. Samples were centrifuged at 2000g for 10 minutes, with serum stored at −80°C until time of use. For complete blood cell counts, un-heparinized whole blood was placed into EDTA Microtainer tubes (BD). Blood counts were performed on a Hemavet HV950 (Drew Scientific) in the In-Vivo Animal Core at the University of Michigan.

### Bronchoalveolar lavage, cell count, and differential

Mice were euthanized ketamine and xylazine. Bronchoalveolar lavage (BAL) was performed as described previously^24^. The trachea was exposed and intubated using a 1.7-mm outer diameter polyethylene catheter. Active insufflation of PBS 5 mM EDTA in 1 ml aliquots was performed, 3 times per mouse. BAL was centrifuged, supernatant removed, aliquoted, and stored at −80 °C until further use. Cell pellets were resuspended and counted using Trypan blue exclusion counting on a hemacytometer. Cytospins were prepared and stained with Diff-Quick (Baxter) to determine differential for polymorphonuclear nuclear (PMN) and mononuclear cells.

### Flow cytometry and cell sorting

BM or BAL cells were washed, blocked for non-specific staining with anti-CD16/32 Fc receptor block (clone 2.4G2, BD), and stained with fluorophore conjugated antibodies prior to analysis on a Attune NxT (ThermoFisher) or MA900 (Sony) flow cytometer and cell sorter. For BM preparations, cells from a single femur and tibia were lineage depleted using biotinylated beads (STEMCell) and biotinylated antibodies against TER-119, CD19, CD3e. Briefly, cells were resuspended in PBS with 1 mM EDTA, 2% FBS, blocked with 5% rat serum, and incubated with biotinylated antibodies for 5 minutes at room temperature. Lineage depletion was performed on an EasyEights EasySep magnet (STEMCell) per protocol. In some studies evaluating BM progenitors, CD16/32 block was substituted with fluorophore conjugated CD16/32 antibody. Anti-bodies included: Ly6G (clone 1A8, Biolegend), CD11b (clone M1/70, Biolegend), CD45 (clone 30-F11, Biolegend), Ly6C (clone HK1.4, Biolegend), CD11c (clone N418, Biolegend), CD64 (clone X54–5/7.1.1, BD), CD24 (clone M1/69, BD), MHCII/I-A/E (clone M5/144.15.2, Biolegend), Siglec-F (E50–2440, BD Pharmingen), CD34 (HM34, Biolegend) CD115 (AFS98, Biolegend), CD135/Flt3 (A2-F10, Biolegend), CD117/c-kit (2B8, Biolegend), CD16/32 (93, Biolegend). Lineage markers TER-119 (TER-119, Biolegend), CD3e (145–2C11, Biolegend), B220 (RA3–6B2, Biolegend), CD19 (eBIO1D3, eBioscience) NK1.1 (PK136, Biolegend). 7-AAD was used for live/dead discrimination. Selected populations were isolated by cell sorting, 13,000 – 300,000 cells directly into TRIzol LS (Invitrogen) for RNA-sequencing (RNAseq) and 50 – 60,000 cells into PBS with 2% BSA (Thermofisher) for ATAC-sequencing (ATACseq).

### Quantification of BAL and supernatant proteins

BAL samples and supernatants were assayed for various proteins using the following ELISAs at dilutions where all samples were within the dynamic range of the standard curve: TNFα, IL-1β, IL-6, MIP-2, MCP-1, KC, IL-1ra, S100A8/A9, RAGE (Mouse Duoset, R&D Systems); Pierce BCA assay (Thermo Scientific); Albumin (Bethyl laboratories).

### RNA Isolation

RNA was isolated from monocyte and progenitor populations using TRIzol LS (Invitrogen) as described by the manufacturer. DNAse treatment was performed on RNeasy Mini Kit columns (Qiagen) prior to sequencing.

### RNA-sequencing and analysis

RNA quality was assessed using a bioanalyzer (Agilent) and all RIN were greater than 7. Library preparation, sequencing, and identification of transcript frequency were performed by the University of Michigan Advanced Genomics Core. Library preparation was performed using the low input SMARTer smRNA-Seq kit (Takara). Samples were subjected to 151bp paired-end sequencing, with an average of 30–40 million reads per sample, according to the manufacturer’s protocol (Illumina NovaSeq). BCL Convert Conversion Software (v3.9.3, Illumina) was used to generate de-multiplexed Fastq files. The reads were trimmed using *Cutadapt* (v2.3)^77^. The reads were evaluated with *FastQC* (v0.11.8, Babraham Bioinformatics) to determine quality of the data. Reads were mapped to reference genome GRCm38 (ENSEMBL 102), using *STAR* (v2.7.8a)^78^ or *HISAT2* (v 2.1.0)^79^. Count estimates performed with *RSEM* (v1.3.3)^80^ or *htseq-count* (version 0.13.5)^81^. Alignment options followed ENCODE standards for RNA-seq. PCA was performed using *prcomp* and a single outlier in the CLP group was excluded from analysis for a total of 4 CLP and 5 unoperated samples (Figure S9). Differential gene expression analysis was conducted with *DESeq2* (version 1.38.3)^82^, followed by pathway analysis using *pathfindR* (v1.6.4)^83^. All code is available online at https://github.com/B-McBean/denstaedt_monocyte. See Genomic Data Supplement for detailed differentially expressed gene lists.

### ATAC-sequencing and analysis

Cell suspensions were brought to the University of Michigan Advanced Genomics Core for library preparation using the Omni-ATAC-Seq protocol^84^. Libraries were cleaned using MinElute (Qiagen) columns and AMPure XP beads (Beckman Coulter) before quantitation with the Qubit HS dsDNA kit (ThermoFisher), and quality assessment on a TapeStation HS D1000 kit (Agilent). The libraries were pooled and quantitated by qPCR using a Library Quantitation Kit – Illumina Platforms (KAPA) before PE-50 sequencing on a NextSeq2000 P3 flow cell with an average of 100 million reads per sample. Sample quality was assessed by *FastQC* (v0.11.8, Babraham Bioinformatics). We aligned reads to mm10 with *HISAT2* (v2.1.0)^79^. Filtering steps are performed with *samtools* (v1.2)^85^ and the parameters: -q 10 -F 4.Unmapped reads and alignments below a MAPQ threshold were removed. Reads in blacklisted regions (ENCODE Blacklist Regions) were removed using *bedtools* (v2.28.0)^86^. *F-Seq2* was used to call sample-wise peaks (v2.0.3)^87^ with flags: -f 0 -p_thr 0.05 -pe. Peaks over all samples are merged with *bedtools,* keeping peaks that occurred in at least 3 samples within a treatment group.. Mitochondrial reads were filtered out in R. Differential peak analysis and pathway enrichment performed using the polyenrich function in *chipenrich* (v2.22.0)^88^. Promoter- and enhancer-specific peaks were determined in R from polyenrich output, <|1000| dist_to_tss and >|1000| dist_to_tss, respectively. Motif enrichment analysis performed using Simple Enrichment Analysis package (*SEA*; v5.5.4)^89^. Finding Individual Motif Occurrences (*FIMO*; v5.5.4) was used to assess AP-1 specific motif enrichment^90^. All code is available online at https://github.com/B-McBean/denstaedt_monocyte.

### Generation of monocyte subset gene signatures for monocytes and their progenitors

Gene sets for MDP- and GMP-derived monocyte-committed progenitors (MP and cMoP, respectively) and Ly6C^hi^ monocyte subsets (G-mono and M-mono, respectively) were defined by a prior ex vivo experiment^31^. To calculate the enrichment of monocyte and progenitor subset gene signature expression in sorted Ly6C^hi^ monocyte (Lin^−^/Ly6G^−^/CD115^+^/CD11b^hi^/Ly6C^hi^/c-Kit^−^/Flt3^−^) and BM progenitor, gene expression data were normalized with *DESeq2* (v1.38.3) and the mean expression level of all genes in each signature was calculated for each sample and log2 transformed. A Wilcoxon test was used to determine the difference between groups using *ggpubr* (v 0.6.0). See Genomic Data Supplement for detailed gene lists.

### Human monocyte subset analysis

Human-mouse conserved monocyte subsets were defined using scRNAseq gene lists from mouse and human lung tumor^60^. Similarly, mouse Neumo and DCmo gene signatures were defined using mouse BM Ly6C^hi^ monocytes^34^. Human peripheral blood monocyte subsets (MS1-MS4) during acute bacterial infection were previously determined^57^. This data was accessed and analyzed through the Single Cell Portal (Broad Institute, https://singlecell.broadinstitute.org/single_cell/study/SCP548/, accessed January 25^th^, 2024) using the gene search and dot plot functions at default settings. Transcripts per million (TPM) counts for specific genes were used from sorted BM Ly6C^hi^ monocytes, as detailed above. Gene TPM counts from CD14+ monocytes from critically-ill patients with sepsis^58^ (GSE139913), and hospitalized patients with community acquired pneumonia^59^ (GSE160329) and their respective healthy controls were acquired using the *Gene Expression Omnibus* web client, GEO2R (NCBI, https://www.ncbi.nlm.nih.gov/geo/geo2r/ accessed January 25^th^, 2024). See Genomic Data Supplement for detailed gene lists.

### Blood count analysis in patients surviving sepsis

The U.S. Veterans Affairs (VA) healthcare system provides comprehensive medical care to over 6 million veterans ^91^. During the study period, VA used a single electronic health record (EHR) system, archived in the Corporate Data Warehouse (CDW) accessible for research^91^. Patient and hospitalization data, including demographics, comorbidities and hospital treatments were extracted from the VA Corporate Data Warehouse (CDW), as described previously^62,92^. Sepsis hospitalizations with live discharge and relevant laboratory data were identified across 138 nationwide VA hospitals (2013 to 2018). Sepsis hospitalizations were identified as previously described^21,62,92–94^ using electronic health record data. Specifically, we identified hospitalizations admitted through the 1) emergency department with evidence of 2) suspected infection and 3) ≥ 1 acute organ dysfunction ^93^ with specific criteria available in Supplemental Table 2. Live discharge was defined as being alive on the calendar day of discharge and the day following (to exclude patients discharged home at end of life). We defined relevant laboratory data as having both absolute monocyte count (AMC) and absolute neutrophil count (ANC) on the calendar day of discharge or day prior. Laboratory values were cleaned and standardized as previously described^21,62^. Non-physiologic laboratory values were excluded (Supplemental Table 3) and then the top and bottom 1% of the remaining values were excluded (Supplemental Table 4).

The association of each parameter with 90-day mortality was evaluated through fitting Cox proportional hazards models and restricted cubic splines using the R package *rms* (v6.0–0). To evaluate the effect of concomitant ANC on the association of AMC with 90-day mortality, the interaction between ANC and AMC was modeled using Cox proportional hazards and the interaction surface was plotted using the R package *visreq* (v2.7.0). All statistical code is available online (https://github.com/CCMRPulmCritCare/MonocyteAnalysis).

### Data analysis

Analyses included ANOVA followed by post-hoc testing when ANOVA was significant or unpaired t testing as indicated in the text. In order to minimize spurious comparisons, we prespecified post-hoc comparisons only between CLP/unoperated. Heatmaps were created using gene TPM counts and were normalized by gene across all samples. All figures show mean and standard error unless otherwise specified. Statistical analyses were carried out in Prism (version 9, Graphpad).

## Supplementary Material

Supplement 1

## Data Availability

Data is available through publicly accessible databases (see Methods), please contact the corresponding author for other data available upon request.

## References

[1] Ifrim D. C. Trained immunity or tolerance: Opposing functional programs induced in human monocytes after engagement of various pattern recognition receptors. Clin. Vaccine Immunol. 21, 534–545 (2014). 24521784 10.1128/CVI.00688-13 PMC3993125

[2] Divangahi M. Trained immunity, tolerance, priming and differentiation: distinct immunological processes. Nature Immunology vol. 22 2–6 (2021). 33293712 10.1038/s41590-020-00845-6 PMC8020292

[3] Lachmandas E. Microbial stimulation of different Toll-like receptor signalling pathways induces diverse metabolic programmes in human monocytes. Nat. Microbiol. 2, 1–10 (2016). 10.1038/nmicrobiol.2016.246 27991883

[4] Dulfer E. A., Joosten L. A. B. & Netea M. G. Enduring echoes: Post-infectious long-term changes in innate immunity. Eur. J. Intern. Med. (2023) doi:10.1016/j.ejim.2023.12.020. 38135583

[5] Denstaedt S. J., Singer B. H. & Standiford T. J. Sepsis and Nosocomial Infection: Patient Characteristics, Mechanisms, and Modulation. Front. Immunol. 9, 2446 (2018). 30459764 10.3389/fimmu.2018.02446 PMC6232897

[6] Foster S. L., Hargreaves D. C. & Medzhitov R. Gene-specific control of inflammation by TLR-induced chromatin modifications. Nature 447, 972–8 (2007). 17538624 10.1038/nature05836

[7] Munoz C. Dysregulation of in vitro cytokine production by monocytes during sepsis. J. Clin. Invest. 88, 1747–1754 (1991). 1939659 10.1172/JCI115493 PMC295719

[8] Hoogeveen R. M. Monocyte and haematopoietic progenitor reprogramming as common mechanism underlying chronic inflammatory and cardiovascular diseases. Eur. Heart J. 1–10 (2017) doi:10.1093/eurheartj/ehx581. 29069365 PMC6174026

[9] Bekkering S. In Vitro Experimental Model of Trained Innate Immunity in Human. 23, 926–933 (2016). 10.1128/CVI.00349-16 PMC5139603 27733422

[10] Quintin J. Candida albicans infection affords protection against reinfection via functional reprogramming of monocytes. Cell Host Microbe 12, 223–232 (2012). 22901542 10.1016/j.chom.2012.06.006 PMC3864037

[11] Dominguez-Andres J. & Netea M. G. Long-term reprogramming of the innate immune system. J. Leukoc. Biol. 1–10 (2018) doi:10.1002/JLB.MR0318-104R. 29999546

[12] McDermott K. W. & Roemer M. Most Frequent Principal Diagnoses for Inpatient Stays in U.S. Hospitals, 2018. HCUP Stat. Br. (2021). 34428003

[13] Rudd K. E. Global, regional, and national sepsis incidence and mortality, 1990–2017: analysis for the Global Burden of Disease Study. Lancet (London, England) 395, 200–211 (2020). 31954465 10.1016/S0140-6736(19)32989-7 PMC6970225

[14] Prescott H. C., Langa K. M. & Iwashyna T. J. Readmission diagnoses after hospitalization for severe sepsis and other acute medical conditions. JAMA 313, 1055–7 (2015). 25756444 10.1001/jama.2015.1410 PMC4760618

[15] Prescott H. C., Osterholzer J. J., Langa K. M., Angus D. C. & Iwashyna T. J. Late mortality after sepsis: propensity matched cohort study. BMJ 353, i2375 (2016). 27189000 10.1136/bmj.i2375 PMC4869794

[16] Shankar-Hari M. Rate and risk factors for rehospitalisation in sepsis survivors: systematic review and meta-analysis. Intensive Care Medicine vol. 46 619–636 (2020). 31974919 10.1007/s00134-019-05908-3 PMC7222906

[17] Donnelly J. P., Wang X. Q., Iwashyna T. J. & Prescott H. C. Readmission and Death after Initial Hospital Discharge among Patients with COVID-19 in a Large Multihospital System. JAMA - J. Am. Med. Assoc. 325, 304–306 (2021). 10.1001/jama.2020.21465 PMC7737131 33315057

[18] Prescott H. C. & Angus D. C. Enhancing recovery from sepsis: A review. JAMA - J. Am. Med. Assoc. 319, 62–75 (2018). 10.1001/jama.2017.17687 PMC5839473 29297082

[19] Yende S. Inflammatory Markers at Hospital Discharge Predict Subsequent Mortality after Pneumonia and Sepsis. (2008) doi:10.1164/rccm.200712-1777OC. PMC2720087 18369199

[20] Yende S. Long-term Host Immune Response Trajectories Among Hospitalized Patients With Sepsis. JAMA Netw. Open 2, e198686 (2019). 10.1001/jamanetworkopen.2019.8686 PMC6686981 31390038

[21] Denstaedt S. J. Blood count derangements after sepsis and association with post-hospital outcomes. Front. Immunol. 14, 1–11 (2023). 10.3389/fimmu.2023.1133351 PMC10018394 36936903

[22] Cheong J. Epigenetic memory of coronavirus infection in innate immune cells and their progenitors. Cell 186, 3882–3902.e24 (2023). 37597510 10.1016/j.cell.2023.07.019 PMC10638861

[23] Prescott H. C. Understanding and Enhancing Sepsis Survivorship. Priorities for Research and Practice. Am. J. Respir. Crit. Care Med. 200, 972–981 (2019). 31161771 10.1164/rccm.201812-2383CP PMC6794113

[24] Denstaedt S. J. Long-term survivors of murine sepsis are predisposed to enhanced LPS-induced lung injury and pro-inflammatory immune reprogramming. Am. J. Physiol. Cell. Mol. Physiol. (2021) doi:10.1152/ajplung.00123.2021. PMC8410111 34161747

[25] Herold S., Mayer K. & Lohmeyer J. Acute Lung Injury: How Macrophages Orchestrate Resolution of Inflammation and Tissue Repair. Front. Immunol. 2, 65 (2011). 22566854 10.3389/fimmu.2011.00065 PMC3342347

[26] Herold S. Exudate Macrophages Attenuate Lung Injury by the Release of IL-1 Receptor Antagonist in Gram-negative Pneumonia. Am. J. Respir. Crit. Care Med. 183, 1380–1390 (2011). 21278303 10.1164/rccm.201009-1431OC

[27] Herold S. Lung epithelial apoptosis in influenza virus pneumonia: The role of macrophage-expressed TNF-related apoptosis-inducing ligand. J. Exp. Med. 205, 3065–3077 (2008). 19064696 10.1084/jem.20080201 PMC2605231

[28] Wolf A. A., Yáñez A., Barman P. K. & Goodridge H. S. The ontogeny of monocyte subsets. Frontiers in Immunology vol. 10 1642 (2019). 31379841 10.3389/fimmu.2019.01642 PMC6650567

[29] Rhee C. Limited plasticity of monocyte fate and function associated with epigenetic scripting at the level of progenitors. Blood 142, 658–674 (2023). 37267513 10.1182/blood.2023020257 PMC10447620

[30] Goodridge H. S. Progenitor diversity defines monocyte roles. Blood 142, 617–619 (2023). 37590027 10.1182/blood.2023021298 PMC10485370

[31] Yáñez A. Granulocyte-Monocyte Progenitors and Monocyte-Dendritic Cell Progenitors Independently Produce Functionally Distinct Monocytes. Immunity 47, 890–902.e4 (2017). 29166589 10.1016/j.immuni.2017.10.021 PMC5726802

[32] Ikeda N. The early neutrophil-committed progenitors aberrantly differentiate into immunoregulatory monocytes during emergency myelopoiesis. CellReports 42, 112165 (2023). 10.1016/j.celrep.2023.112165 36862552

[33] Weinreb C. & , Alejo Rodriguez-Fraticelli, Camargo Fernando D., A. M. K. State To Fate During Differentiation. Science (80-. ). 367, 755 (2020). 10.1126/science.aaw3381 PMC7608074 31974159

[34] Barman P. K. Production of MHCII-expressing classical monocytes increases during aging in mice and humans. Aging Cell 00, e13701 (2022). 10.1111/acel.13701 PMC9577948 36040389

[35] Larsen S. B. Establishment, maintenance, and recall of inflammatory memory. Cell Stem Cell 28, 1758–1774.e8 (2021). 34320411 10.1016/j.stem.2021.07.001 PMC8500942

[36] Naik S. Inflammatory memory sensitizes skin epithelial stem cells to tissue damage. Nature 550, 475–480 (2017). 29045388 10.1038/nature24271 PMC5808576

[37] Cirovic B. BCG Vaccination in Humans Elicits Trained Immunity via the Hematopoietic Progenitor Compartment. Cell Host Microbe 28, 322–334.e5 (2020). 32544459 10.1016/j.chom.2020.05.014 PMC7295478

[38] Jentho E. Trained innate immunity, long-lasting epigenetic modulation, and skewed myelopoiesis by heme. Proc. Natl. Acad. Sci. U. S. A. 118, 1–10 (2021). 10.1073/pnas.2102698118 PMC8545490 34663697

[39] Christ A. Western Diet Triggers NLRP3-Dependent Innate Immune Reprogramming. Cell 172, 162–175.e14 (2018). 29328911 10.1016/j.cell.2017.12.013 PMC6324559

[40] Kalafati L. Innate Immune Training of Granulopoiesis Promotes Anti-tumor Activity. Cell 183, 771–785.e12 (2020). 33125892 10.1016/j.cell.2020.09.058 PMC7599076

[41] Li X. Maladaptive innate immune training of myelopoiesis links inflammatory comorbidities. Cell 185, 1709–1727.e18 (2022). 35483374 10.1016/j.cell.2022.03.043 PMC9106933

[42] Mitroulis I. Modulation of Myelopoiesis Progenitors Is an Integral Component of Trained Immunity. Cell 172, 147–161.e12 (2018). 29328910 10.1016/j.cell.2017.11.034 PMC5766828

[43] Niec R. E., Rudensky A. Y. & Fuchs E. Inflammatory adaptation in barrier tissues. Cell 184, 3361–3375 (2021). 34171319 10.1016/j.cell.2021.05.036 PMC8336675

[44] de Laval B. C/EBPβ-Dependent Epigenetic Memory Induces Trained Immunity in Hematopoietic Stem Cells. Cell Stem Cell 26, 657–674.e8 (2020). 32169166 10.1016/j.stem.2020.01.017

[45] Uchida T. Receptor for Advanced Glycation End-Products Is a Marker of Type I Cell Injury in Acute Lung Injury. Am. J. Respir. Crit. Care Med. 173, 1008–1015 (2006). 16456142 10.1164/rccm.200509-1477OC PMC2662912

[46] Rittirsch D. Acute Lung Injury Induced by Lipopolysaccharide Is Independent of Complement Activation. J. Immunol. 180, 7664–7672 (2008). 18490769 10.4049/jimmunol.180.11.7664 PMC2753408

[47] Chignard M. & Balloy V. Neutrophil recruitment and increased permeability during acute lung injury induced by lipopolysaccharide. Am. J. Physiol. - Lung Cell. Mol. Physiol. 279, 1083–1090 (2000). 10.1152/ajplung.2000.279.6.L1083 11076798

[48] Grommes J. & Soehnlein O. Contribution of neutrophils to acute lung injury. Mol. Med. 17, 293–307 (2011). 21046059 10.2119/molmed.2010.00138 PMC3060975

[49] Sprenkeler E. G. G. S100A8/A9 Is a Marker for the Release of Neutrophil Extracellular Traps and Induces Neutrophil Activation. Cells 2022, Vol. 11, Page 236 11, 236 (2022). 35053354 10.3390/cells11020236 PMC8773660

[50] Stroncek D. F., Shankar R. A. & Skubitz K. M. The subcellular distribution of myeloid-related protein 8 (MRP8) and MRP14 in human neutrophils. J. Transl. Med. 3, (2005). 10.1186/1479-5876-3-36 PMC1253536 16191197

[51] Sreejit G. Neutrophil-Derived S100A8/A9 Amplify Granulopoiesis after Myocardial Infarction. Circulation 141, 1080–1094 (2020). 31941367 10.1161/CIRCULATIONAHA.119.043833 PMC7122461

[52] Simard J.-C., Girard D. & Tessier P. A. Induction of neutrophil degranulation by S100A9 via a MAPK-dependent mechanism. J. Leukoc. Biol. 87, 905–914 (2010). 20103766 10.1189/jlb.1009676

[53] Sajti E. Transcriptomic and epigenetic mechanisms underlying myeloid diversity in the lung. Nat. Immunol. 21, 221–231 (2020). 31959980 10.1038/s41590-019-0582-z PMC7667722

[54] Yáñez A. & Goodridge H. S. Identification and isolation of oligopotent and lineage-committed myeloid progenitors from mouse bone marrow. J. Vis. Exp. 2018, 1–9 (2018). 10.3791/58061 PMC6126592 30102291

[55] Patel A. A. The fate and lifespan of human monocyte subsets in steady state and systemic inflammation. J. Exp. Med. 1–11 (2017) doi:10.1084/jem.20170355. PMC5502436 28606987

[56] Yona S. Fate mapping reveals origins and dynamics of monocytes and tissue macrophages under homeostasis. Immunity 38, 79–91 (2013). 23273845 10.1016/j.immuni.2012.12.001 PMC3908543

[57] Reyes M. An immune-cell signature of bacterial sepsis. Nat. Med. 1–8 (2020) doi:10.1038/s41591-020-0752-4. 32066974 PMC7235950

[58] Liepelt A. Differential gene expression in circulating CD14+ monocytes indicates the prognosis of critically ill patients with sepsis. J. Clin. Med. 9, 1–22 (2020). 10.3390/jcm9010127 PMC7019484 31906585

[59] Brands X. An epigenetic and transcriptomic signature of immune tolerance in human monocytes through multi-omics integration. Genome Med. 13, 1–17 (2021). 34399830 10.1186/s13073-021-00948-1 PMC8365568

[60] Zilionis R. Single-Cell Transcriptomics of Human and Mouse Lung Cancers Reveals Conserved Myeloid Populations across Individuals and Species. Immunity 50, 1317–1334.e10 (2019). 30979687 10.1016/j.immuni.2019.03.009 PMC6620049

[61] Prescott H. C., Langa K. M. & Iwashyna T. J. Readmission diagnoses after hospitalization for severe sepsis and other acute medical conditions. JAMA - J. Am. Med. Assoc. 313, 1055–1057 (2015). 10.1001/jama.2015.1410 PMC4760618 25756444

[62] Wang X. Q. Veterans Affairs patient database (VAPD 2014–2017): Building nationwide granular data for clinical discovery. BMC Med. Res. Methodol. 19, 1–9 (2019). 31068135 10.1186/s12874-019-0740-x PMC6505066

[63] Millar J. E. The genomic landscape of Acute Respiratory Distress Syndrome : a meta-analysis by information content of genome-wide studies of the host response . medRxiv (2024) doi:10.1101/2024.02.13.24301089.

[64] Parkos C. A. Neutrophil-Epithelial Interactions A Double-Edged Sword. Am. J. Pathol. 186, 1404–1416 (2016). 27083514 10.1016/j.ajpath.2016.02.001 PMC4901132

[65] Trzebanski S. & Jung S. Plasticity of monocyte development and monocyte fates. Immunol. Lett. 227, 66–78 (2020). 32814154 10.1016/j.imlet.2020.07.007

[66] Leite G. G. F. Monocyte state 1 (MS1) cells in critically ill patients with sepsis or non-infectious conditions: association with disease course and host response. Crit. Care 28, 1–15 (2024). 38504349 10.1186/s13054-024-04868-5 PMC10953179

[67] Ikeda N. Emergence of immunoregulatory Ym1+Ly6Chi monocytes during recovery phase of tissue injury. Sci. Immunol. 3, (2018). 10.1126/sciimmunol.aat0207 30291130

[68] Basso E. K. G. Immunoregulatory and neutrophil - like monocyte subsets with distinct single - cell transcriptomic signatures emerge following brain injury. J. Neuroinflammation 1–17 (2024) doi:10.1186/s12974-024-03032-8. 38310257 PMC10838447

[69] Li W. A single-cell view on host immune transcriptional response to in vivo BCG-induced trained immunity. Cell Rep. 42, 112487 (2023). 37155329 10.1016/j.celrep.2023.112487 PMC10242447

[70] Cheng S.-C. mTOR- and HIF-1 -mediated aerobic glycolysis as metabolic basis for trained immunity. Science (80-. ). 345, 1250684–1250684 (2014). 10.1126/science.1250684 PMC4226238 25258083

[71] Denstaedt S. J. Persistent Neuroinflammation and Brain-Specific Immune Priming in a Novel Survival Model of Murine Pneumosepsis. Shock 54, 78–86 (2020). 31415473 10.1097/SHK.0000000000001435 PMC7015772

[72] Denstaedt S. J. S100A8/A9 Drives Neuroinflammatory Priming and Protects against Anxiety-like Behavior after Sepsis. J. Immunol. 200, 3188–3200 (2018). 29563178 10.4049/jimmunol.1700834 PMC5915914

[73] Wolf A. A., Yáñez A., Barman P. K. & Goodridge H. S. The ontogeny of monocyte subsets. Front. Immunol. 10, 1642 (2019). 31379841 10.3389/fimmu.2019.01642 PMC6650567

[74] Dickson R. P. Enrichment of the lung microbiome with gut bacteria in sepsis and the acute respiratory distress syndrome. Nat. Microbiol. 1, 1 (2016). 10.1038/nmicrobiol.2016.113 PMC5076472 27670109

[75] Jochen Pfirstinger S. Mice Chemokine Receptors CCR2 and CCR5 in Expression and Characterization of the. J Immunol Ref. 166, 4697–4704 (2001). 10.4049/jimmunol.166.7.4697 11254730

[76] Swamydas M., Luo Y., Dorf M. E. & Lionakis M. S. Isolation of mouse neutrophils. Curr. Protoc. Immunol. 2015, 3.20.1–3.20.15 (2015). 10.1002/0471142735.im0320s110 PMC4574512 26237011

[77] Martin M. Cutadapt removes adapter sequences from high-throughput sequencing reads. EMBnet.journal 17, 10 (2011).

[78] Dobin A. STAR: Ultrafast universal RNA-seq aligner. Bioinformatics 29, 15–21 (2013). 23104886 10.1093/bioinformatics/bts635 PMC3530905

[79] Kim D., Paggi J. M., Park C., Bennett C. & Salzberg S. L. Graph-based genome alignment and genotyping with HISAT2 and HISAT-genotype. Nat. Biotechnol. 37, 907– 915 (2019). 31375807 10.1038/s41587-019-0201-4 PMC7605509

[80] Li B. & Dewey C. N. RSEM: accurate transcript quantification from RNA-Seq data with or without a reference genome. BMC Bioinformatics 12, 323 (2011). 21816040 10.1186/1471-2105-12-323 PMC3163565

[81] Anders S., Pyl P. T. & Huber W. HTSeq-A Python framework to work with high-throughput sequencing data. Bioinformatics 31, 166–169 (2015). 25260700 10.1093/bioinformatics/btu638 PMC4287950

[82] Love M. I., Huber W. & Anders S. Moderated estimation of fold change and dispersion for RNA-seq data with DESeq2. Genome Biol. 15, 550 (2014). 25516281 10.1186/s13059-014-0550-8 PMC4302049

[83] Ulgen E., Ozisik O. & Sezerman O. U. PathfindR: An R package for comprehensive identification of enriched pathways in omics data through active subnetworks. Front. Genet. 10, 425394 (2019). 10.3389/fgene.2019.00858 PMC6773876 31608109

[84] Corces M. R. An improved ATAC-seq protocol reduces background and enables interrogation of frozen tissues. Nat. Methods 14, 959–962 (2017). 28846090 10.1038/nmeth.4396 PMC5623106

[85] Li H. The Sequence Alignment/Map format and SAMtools. Bioinformatics 25, 2078–2079 (2009). 19505943 10.1093/bioinformatics/btp352 PMC2723002

[86] Quinlan A. R. & Hall I. M. BEDTools: A flexible suite of utilities for comparing genomic features. Bioinformatics 26, 841–842 (2010). 20110278 10.1093/bioinformatics/btq033 PMC2832824

[87] Zhao N. & Boyle A. P. F-Seq2: Improving the feature density based peak caller with dynamic statistics. NAR Genomics Bioinforma. 3, 1–8 (2021). 10.1093/nargab/lqab012 PMC7902237 33655209

[88] Lee C. T. Poly-Enrich: count-based methods for gene set enrichment testing with genomic regions. NAR Genomics Bioinforma. 2, 1–13 (2020). 10.1093/nargab/lqaa006 PMC7003681 32051932

[89] Bailey T. L. & Grant C. E. SEA: Simple Enrichment Analysis of motifs. bioRxiv 2021.08.23.457422 (2021) doi:10.1101/2021.08.23.457422.

[90] Grant C. E., Bailey T. L. & Noble W. S. FIMO: Scanning for occurrences of a given motif. Bioinformatics 27, 1017–1018 (2011). 21330290 10.1093/bioinformatics/btr064 PMC3065696

[91] Fihn S. D. Insights from advanced analytics at the Veterans Health Administration. Health Aff. (Millwood). 33, 1203–1211 (2014). 25006147 10.1377/hlthaff.2014.0054

[92] Vincent B. M., Wiitala W. L., Burns J. A., Iwashyna T. J. & Prescott H. C. Using Veterans Affairs Corporate Data Warehouse to identify 30-day hospital readmissions. Heal. Serv. Outcomes Res. Methodol. 18, 143–154 (2018). 10.1007/s10742-018-0178-3 PMC9518959 36176573

[93] Wayne M. T. Measurement of Sepsis in a National Cohort Using Three Different Methods to Define Baseline Organ Function. Ann. Am. Thorac. Soc. 18, 648–655 (2021). 33476245 10.1513/AnnalsATS.202009-1130OC PMC8008999

[94] Wayne M. T. Temporal Trends and Hospital Variation in Time-to-Antibiotics Among Veterans Hospitalized With Sepsis. JAMA Netw. open 4, (2021). 10.1001/jamanetworkopen.2021.23950 PMC8424480 34491351

